# Ascorbic acid induced HepG2 cells' apoptosis via intracellular reductive stress: Erratum

**DOI:** 10.7150/thno.56023

**Published:** 2021-01-01

**Authors:** Xiaonan Gao, Keyan Wei, Bo Hu, Kehua Xu, Bo Tang

**Affiliations:** College of Chemistry, Chemical Engineering and Materials Science, Collaborative Innovation Center of Functionalized Probes for Chemical Imaging in Universities of Shandong, Key Laboratory of Molecular and Nano Probes, Ministry of Education, Shandong Provincial Key Laboratory of Clean Production of Fine Chemicals, Shandong Normal University, Jinan 250014, P. R. China

The authors regret that the images in the original version of Figure 5A (II) Day 8, the inserted picture of the mouse was chosen by mistake and misused by accident previously. The correct version of Figure 5A is shown here. This correction does not influence any of the experimental results and discussion or the conclusions reported in the article.

## Figures and Tables

**Figure 5:**
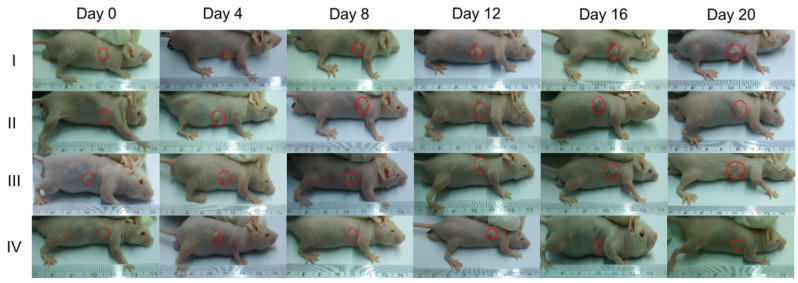
Antitumor effect of CTMP-AA: (A) the representative images of mice following 20-days treatment for each treatment group.

